# Oxidative Stress, Antioxidants, Physical Activity, and the Prevention of Breast Cancer Initiation and Progression

**DOI:** 10.15436/2378-6841.18.2013

**Published:** 2018-11-07

**Authors:** Steven S. Coughlin

**Affiliations:** 1 Department of Population Health Sciences, Medical College of Georgia, Augusta University, Augusta, GA; 2 Research Service, Charlie Norwood Veterans Administration Medical Center, Augusta, GA

## Introduction

Oxidative stress has a role in the initiation and progression of breast cancer (Jezierska-Drutel, A., et al 2013). Oxidative stress results from an imbalance between antioxidants and unstable reactive oxygen and nitrogen species (superoxide anion, hydrogen peroxide, reactive nitrogen species) that lack one or more unpaired electrons (Jezierska-Drutel, A., et al 2013). Oxidative stress occurs when antioxidants are unable to scavenge excess oxygen free radicals (Zarrini, A.S., et al 2016). Redox imbalance contributes to cancer and other chronic diseases. The activity of reactive oxygen species on DNA, proteins, and lipids promotes changes in cell physiology that can interfere with its normal functioning (Panis, C., et al 2016). High levels of reactive oxygen species disrupt cellular processes by nonspecifically attacking DNA, proteins, and lipids (Schumacker PT., 2015). Lower levels of reactive oxygen species can act as cell signaling messengers by reversibly oxidizing protein thiol groups and modifying protein structure.

By damaging DNA, activating oncogenes, and producing genomic instability, reactive oxygen species can initiate carcinogenesis (Toyokuni S., et al., 1995). Reactive oxygen species such as superoxide anion, hydrogen peroxide, and hydroxyl radical influence cell signaling and affect a number of molecular pathways related to cell proliferation, angiogenesis, invasion of surrounding tissues, and the metastatic process (Hecht F., et al. 2016). Although some oxidants contribute to mutation and growth, excessive oxidative stress can slow proliferation and damage cancer cells (Schumacker PT., 2015).

Breast cancer tumors with enhanced proliferative capacity produce high levels of reactive oxygen species during their chronic cycles of ischemia, reperfusion, and angiogenesis, resulting in growth signaling (Panis, C., et al 2016). Cancer cells can induce oxidative damage in surrounding normal tissues. Higher levels of circulating malondialdehyde has been reported in advanced stages of breast cancer than in early stages, suggesting stage differences in oxidative stress (Zarrini, A.S., et al 2016). Breast cancer patients have been found to have higher levels of malondialdehyde, a peroxidation product and marker of oxidative stress, than control patients (Ray G, et al. 2000; Yeh C.C., et al. 2005; Sheeba C., et al. 2010; Zarrini, A.S., et al 2016).

Many chemotherapeutic drugs and radiation treatments eliminate tumors through the induction of reactive oxygen species in cancer cells (Kawahara B., et al. 2017). However, compared to normal cells, cancer cells have an increased antioxi-dative capacity that can lead to resistance to chemotherapeutic drugs. Cancer cells have an increased antioxidant potential due to an increased ratio of reduced to oxidized glutathione (Conklin KA., 2004; Kawahara B., et al. 2017).

Endogenous antioxidants, including non-enzyme and enzyme antioxidant defense systems (e.g., reduced glutathione, superoxide dismutase, catalase, and glutathione peroxidase) act to prevent or limit tissue damage by scavenging free radicals in cells (Schumacker PT., 2015; Ramirez-Exposito MJ., et al 2017). Superoxide dismutase metabolizes free radicals and dismutates superoxide anions to H2O2, thereby protecting cells from lipid peroxidation (Ramirez-Exposito MJ., et al 2017). Catalase converts H2O2 into H2O and O2.

Exogenous antioxidants from dietary intake such as vitamin A, vitamin E, and Vitamin C, may also have a role in the alleviation of oxidative stress, which could reduce risk of breast cancer or slow progression of the disease (Skouroliakou M., et al. 2018). However, no association was observed in the Women’s Health Initiative between multivitamin use and risk of breast cancer (Neuhouser ML., et al. 2009). Chemopreventive agents are increasingly identified for further development based upon biological mechanisms (for example, scavenging of free radicals, antioxidant activity) (Steward WP., et al. 2013). Folate was identified as a potential chemopreventive agent based upon dietary studies and its role in DNA repair (WCRF/AICR, 1997; Potter JD, 2014). However, in the Women’s Antioxidant and Folic Acid Cardiovascular Study, no reduction in breast cancer or total cancer was observed (Zhang SM, et al. 2008).

Results from observational epidemiologic studies conducted over the past several decades demonstrate that physical activity and weight control are important determinants of breast cancer risk and progression (Meyskens FL, et al. 2016). Oxidative stress has been examined in studies of breast cancer and physical activity (Tomasello B, et al 2017). Physical activity increases the production of reactive oxygen species and, due to an adaptation that occurs over time, exercise increases antioxidant capabilities and counters oxidative insults (Acharya A., et al. 2010). It has been hypothesized that oxidative damage markers can be positively impacted by exercise training through enhanced DNA damage repair mechanisms (Soares JP., et al. 2015). The balance of oxidative stress factors is mainly determined by enzymatic mechanisms although exogenous factors such as physical activity and dietary intake can also play an important role (Klaunig JE., et al. 20041; Friedenreich CM., et al. 2016). Exercise training increases oxidative damage repair enzyme capacity and reduces oxidative damage.

In order to inform future studies of antioxidant chemo-preventive agents that may reduce risk of breast cancer initiation or progression, the complexities of how redox imbalance contributes to cancer and other chronic diseases should be considered. This includes the observation that although high levels of reactive oxygen species disrupt cellular processes, lower levels of reactive oxygen species can act as cell signaling messengers by reversibly oxidizing protein thiol groups and modifying protein structure (Schumacker PT., 2015). Thus, there are likely to be dose-response effects of endogenous and exogenous antioxidants. In considering the effects of antioxidants and exercise on breast cancer progression and survival, stage at cancer diagnosis may be important, as higher levels of markers of oxidative stress have been observed in advanced stages of breast cancer than in early stages (Zarrini, A.S., et al 2016). The complexity and genetic heterogeneity of advanced breast cancer suggests that the focus of cancer prevention efforts should be on the early interruption of the carcinogenic process (Meyskens FL, et al. 2016).
